# The silent threat: investigating sleep disturbances in hospitalized patients

**DOI:** 10.1093/intqhc/mzae042

**Published:** 2024-05-10

**Authors:** Corey Adams, Reema Harrison, Anthony Schembri, Moira Junge, Ramesh Walpola

**Affiliations:** Australian Institute of Health Innovation (AIHI), Macquarie University, Sydney, NSW 2113, Australia; Australian Institute of Health Innovation (AIHI), Macquarie University, Sydney, NSW 2113, Australia; St Vincent’s Health Network, Sydney, NSW 2010, Australia; Sleep Health Foundation, Australia; School of Health Sciences, University of New South Wales (UNSW), Sydney, NSW 2052, Australia

**Keywords:** hospitals, sleep, patient experience, nursing, noise, survey, quality, improvement

## Abstract

Sleep disruptions in the hospital setting can have adverse effects on patient safety and well-being, leading to complications like delirium and prolonged recovery. This study aimed to comprehensively assess the factors influencing sleep disturbances in hospital wards, with a comparison of the sleep quality of patients staying in single rooms to those in shared rooms. A mixed-methods approach was used to examine patient-reported sleep quality and sleep disruption factors, in conjunction with objective noise measurements, across seven inpatient wards at an acute tertiary public hospital in Sydney, Australia. The most disruptive factor to sleep in the hospital was noise, ranked as ‘very disruptive’ by 20% of patients, followed by acute health conditions (11%) and nursing interventions (10%). Patients in shared rooms experienced the most disturbed sleep, with 51% reporting ‘poor’ or ‘very poor’ sleep quality. In contrast, only 17% of the patients in single rooms reported the same. Notably, sound levels in shared rooms surpassed 100 dB, highlighting the potential for significant sleep disturbances in shared patient accommodation settings. The results of this study provide a comprehensive overview of the sleep-related challenges faced by patients in hospital, particularly those staying in shared rooms. The insights from this study offer guidance for targeted healthcare improvements to minimize disruptions and enhance the quality of sleep for hospitalized patients.

## Introduction

Sleep is crucial for maintaining overall health and well-being; however, patients frequently report difficulty sleeping in the hospital setting [1–3]. Research indicates that patients sleep for an average of 5 hrs per night in the hospital [3], which is considerably less than their typical sleep duration at home [1]. Additionally, sleep in hospital is often fragmented, preventing patients from reaching deeper and more restorative sleep stages essential for recovery and healing [2]. Impaired sleep can increase a person’s risk of various health complications, including hypertension, impaired glucose tolerance, and increased stress hormone responses [4]. Furthermore, sleep deficiency can also lead to attention and memory lapses, depressive mood, and cognitive impairments, including delirium [1, 5], and has been associated with a heightened susceptibility to healthcare-associated infections, potentially impeding patient recovery and healing [6].

The sleep quality of patients in hospital may be impacted by a multitude of factors, including clinical interactions involving the patient or co-located patients (e.g. nurses conducting patient observations), ambient noise, and exposure to artificial lighting [1, 7]. Noise, in particular, has been identified as a primary cause of sleep disturbances in hospital settings [1, 8, 9]. The hospital environment is typically not conducive to promoting sleep. For instance, the World Health Organization (WHO) advises that sound levels in ward rooms should not surpass 30 dB, while the United States Environmental Protection Agency (EPA) recommends maintaining sound levels below 45 dB during the daytime and 35 dB at night [10]. However, sound levels in clinical areas often exceed these recommendations [11]. Hospitals are notorious for their noisy environments, impacted by various sources such as alarms, paging systems, and medical equipment [12]. These excessive sound levels not only negatively affect patients’ experiences in hospitals but also contribute to annoyance, stress, and potential burnout among hospital staff [8, 13].

To optimize patient health and recovery, sleep assessments should be routinely incorporated into clinical evaluations [14]. However, research indicates a significant underutilization of assessment tools for evaluating sleep in hospitals. For instance, a study has identified that staff infrequently discuss sleep with patients and do not perform formal sleep assessments or use assessment tools to guide nursing interventions [14]. A survey of nursing staff also revealed that patient sleep documentation is primarily based on nursing observations, with minimal use of formal sleep assessment tools in hospitals [15]. Furthermore, healthcare staff may have limited knowledge about sleep issues in hospitals [8], with staff often overestimating patients’ sleep duration and quality in comparison to patients’ reports and biophysiological measures [1]. Research involving clinicians from 15 acute-care hospitals found that there was no current training on sleep care for patients, despite nurses acknowledging its importance and relevance to patient care [15]. Thus, there is an urgent need for improved tools to assess sleep quality and identify sleep disruption causes, enabling more effective sleep management and improvement [14, 15].

Sleep disruption is a significant threat to patient health and safety; yet this issue remains largely overlooked in the hospital setting. Identification of the factors that disrupt sleep in hospital environments may help to inform targeted improvement initiatives to enhance patient sleep and recovery. This study aimed to investigate the factors contributing to sleep disruptions for patients in hospitals, with a comparative analysis between those staying in single rooms and those in shared rooms. Additionally, the study examined the correlation between sleep quality and patient experience ratings.

## Methods

Using observational research design, a multi-methods approach was employed that incorporated patient-reported sleep measures and environmental noise measurement. This research method provided in-depth analysis of subjective sleep experiences, coupled with objective noise measurement, to support targeted interventions. The study was reported in accordance with the Checklist for Reporting Results of Internet E-Surveys (CHERRIES) guidelines.

### Participants

The study was conducted in an acute care, tertiary public hospital located in Sydney, Australia. Eligible participants were patients admitted to any of the seven inpatient wards during the 4-week study period from November to December 2021. To qualify for inclusion, participants needed to be adults (aged ≥18 years) and hospitalized for a minimum of 24 hrs. Exclusion criteria were non-admission to the hospital, hospital stays shorter than 24 hrs, and admission to the Intensive Care Unit. Participation in the study was voluntary and anonymous.

### Instrumentation

#### Patient survey

The ‘Sleep Disruption in Hospitals’ survey [16] was utilized to assess patients’ self-reported experiences of sleep quality while staying in a hospital. This survey consisted of five questions that focused on the overall sleep quality rating and a comparison of sleep quality in the hospital to that at home. Participants were asked to rate the degree of sleep disruption caused by various factors, such as noise, light, clinical interventions, and existing or acute health conditions, using a 5-point Likert scale that ranged from ‘Not at all’ to ‘Very Disruptive’. Additionally, patients were asked to evaluate the overall hospital experience using a 5-point Likert scale, from ‘Very Poor’ to ‘Very Good.’ Before its application in the study, a panel of five consumer representatives reviewed the patient survey to ensure clarity and ease of understanding for respondents.

#### Noise measurement

Casella DBadge2 Noise Badge Dosimeters were utilized for the measurement of sound, which determines the A-weighted frequency, representing the relative loudness as perceived by the human ear [17]. The dosimeters were used to measure both average and peak sound levels, such as LAEQ (the average sound level with A-weighted frequency weighting) and LAFmax (the maximum sound level with A-weighted frequency weighting).

### Procedure

#### Patient survey

The process of distributing the Sleep disruption in Hospitals survey adhered to standard hospital procedures for evaluating patient experiences. Patients received a text message containing a survey link within two weeks after being discharged from the hospital. Patients received a text message containing a survey link within two weeks after being discharged from the hospital, which was disseminated using the Qualtrics platform.

#### Noise measurement

The environmental assessment of ward sound levels was conducted over a 12-hr period (from 8 p.m. to 8 a.m.) in three selected wards. To ensure reliable and accurate data, dosimeters were allocated randomly to both single and shared rooms using a random number generator (Research Randomizer). Patients who were in single rooms with infectious precautions, such as COVID-19, were excluded from the study due to infection control considerations. The dosimeters were calibrated immediately before measurement to maximize accuracy. To approximate the sound perception of patients during their stay, the dosimeters were positioned on the wall, ∼1 m from the patient bedhead, and sound data was logged at one-second intervals.

#### Data analysis

For the patient survey, descriptive statistics and chi-squared analysis methods were employed to analyze the survey data, while Spearman’s rank correlation was computed to assess the relationship between sleep quality and patient experience. Data analysis for the patient survey and noise level measurement was conducted using IBM SPSS Statistics (Version 25).

### Ethical considerations

Ethical approval was provided by the hospital Human Research Ethics Committee (HREC) in Sydney, Australia (2021/ETH11585). Participation was voluntary and participants implied their consent by completing the online survey.

## Results

During the 4-week study period, 694 patients were sent the survey, which was completed by 140 respondents (20.2% response rate). Ten respondents did not complete the questions about their sleep quality, so these participants were removed from the sample. In total, 130 responses were collected and analyzed (mean age 58.2 yrs; range 19–91 yrs) with 52% females (as shown in Table 1). The average length of stay (LOS) was 5.4 days, and the majority of patients (69%, *N* = 90) slept in a shared room, which accommodated up to four patients.

**Table 1.** Participant demographics.

Characteristic	*N* = 130
Sex	
Male	62 (48%)
Female	68 (52%)
Age group (years)	
<20	1 (0.7%)
20–39	21 (16.1%)
40–59	39 (30%)
60–79	53 (40.7%)
≥80	16 (12.3%)
Hospital room	
Single	40 (31%)
Shared	90 (69%)

As shown in Table 2, 38% (49/130) of patients rated sleep in hospital as ‘good’ or ‘very good’ while 40% (52/130) of the patients reported that the quality of their sleep in the hospital setting was either ‘poor’ or ‘very poor’. When compared to their usual sleep at home, 47% (61/130) of patients rated their sleep in the hospital as ‘much worse’. Statistical analysis identified no association between the quality of sleep and either the age or length of stay of the patients surveyed.

**Table 2.** Patient-reported sleep quality in hospital.

	Very poor	Poor	Fair	Good	Very good
Total (130)	27 (21%)	25 (19%)	29 (22%)	35 (27%)	14 (11%)
Single room (41)	3 (7%)	4 (10%)	9 (22%)	16 (41%)	8 (20%)
Shared room (89)	24 (27%)	21 (24%)	20 (22%)	18 (20%)	6 (7%)

### Causes of sleep disturbance in the hospital

During the process of data analysis, scoring was obtained by assigning numerical values to the responses (Not at all = 1, to Very Disruptive = 5) and calculating the average score for the domain. As shown in Table 3, the highest score for disruption was noise (M 3.02, SD 1.37), which was the only factor with a mean score above 3 (i.e. above mid-range). The next most disruptive factors were acute medical condition (M 2.47, SD 1.28) and nursing interventions (M 2.42, SD 1.20), while light was less disruptive (M 2.28, SD 1.25).

**Table 3.** Patient ratings of sleep disruption factors.

Factor	Mean (SD)
Noise	3.02 (1.37)
Acute medical condition	2.47 (1.28)
Nursing interventions	2.42 (1.20)
Light	2.28 (1.25)
Treatments	2.20 (1.17)
Existing medical condition	1.86 (1.27)
Diagnostic tests	1.63 (1.10)
Personal care	1.36 (0.82)

Additionally, analysis identified that *noise* was reported as ‘very disruptive’ by 20% of patients (26/130), followed by *acute conditions* (11%, 14/130) and *nursing interventions* (10%, 13/130). Conversely, *light* was reported as ‘very disruptive’ by only 7% of patients (9/130).

### Sleep quality and overall hospital experience

Spearman’s rank correlation analysis was conducted to examine the relationship between sleep quality and patient experience, which revealed a positive correlation between these two variables, *r* (130) = 0.513, *P* = .01. This finding suggests that there is a moderately strong positive association between patients’ sleep quality ratings and their overall hospital experience.

### Comparing sleep quality in single and shared rooms

A notable difference in sleep quality scores was observed between patients in shared rooms and those in single rooms. Patients in shared rooms reported ‘very poor’ sleep quality almost three times more frequently (27%, 24/89) compared to patients in single rooms (7%, 3/41). Furthermore, a larger percentage of patients in single rooms (61%, 24/41) rated their sleep quality as either ‘good’ or ‘very good’, in contrast to only 27% (24/89) of patients in shared rooms.

Hospital noise emerged as the primary factor contributing to this disparity, which was especially problematic for patients in shared rooms. Noise was rated as ‘quite’ or ‘very disruptive’ by 47% (42/89) of patients in shared rooms, as opposed to 17% (7/41) in single rooms. A chi-squared test of independence revealed a statistically significant relationship between the type of hospital room and noise, χ^2^ (4, *N* = 120) = 13.28, *P* = .01. No significant associations were found between room type and other factors, such as light (*P* = .443), staff checking vital signs (*P* = .505), treatment (*P* = .849), diagnostic tests (*P* = .972), personal care (*P* = .529), existing medical conditions (*P* = .566), and acute medical conditions (*P* = .525).

### Measurement of ward sound levels

#### Noise intensity (average and peak levels)

Overnight, the average sound level for all six wards was 47.2 dB, ranging from 44.7 dB to 49.9 dB. During the 12-hr time period, maximum sound levels (LAFMax) for each room varied from 93.6 to 106.9 dB. The average sound levels (using LAEQ) were higher in the shared rooms (49.9 dB) than single rooms (44.7 dB), as shown in Table 4.

**Table 4.** Sound-level measurements for single and shared rooms.

Location	Room type	LAEQ (av.), dB	LAFMax (av.), dB	LAFMax (max.), dB
Ward A	Single	44.7	46.3	98.4
	Shared	46.5	48.3	102.3
Ward B	Single	47.1	49.1	97.7
	Shared	49.9	51.5	100.5
Ward C	Single	45.5	47.1	93.6
	Shared	49.3	50.3	106.9

Moreover, it is noteworthy that all shared rooms exhibited peak sound levels surpassing 100 decibels (LAFMax) during the night, whereas this did not occur in any of the single rooms.

#### Frequency of sound peaks

The evaluation of sound levels involved an assessment of variations over a 12-hr period overnight, revealing that shared rooms displayed two to three times the number of noise peaks (i.e. peaks surpassing 70 dB) as single rooms (as depicted in Table 5).

**Table 5.** Number of sound-level peaks in single and shared rooms.

	Single	Shared
Ward A	14 peaks	47 peaks
Ward B	31 peaks	60 peaks
Ward C	33 peaks	86 peaks

**Figure 1:**
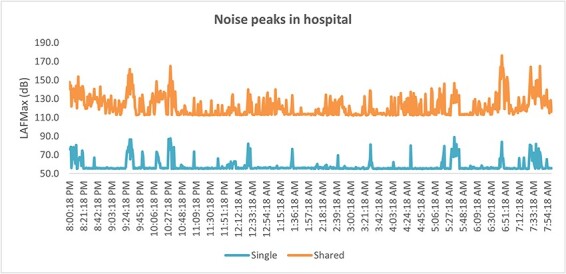
Sound-level peaks in shared and single rooms (example from Ward 3).

The noise patterns were graphed over time (8 p.m. to 8 a.m.), which identified that these sound-level peaks tended to occur frequently throughout the night in shared rooms and intermittently in single rooms (see Fig. 1).

## Discussion

### Statement of principal findings

The findings identified that 40% of patients reported poor or very poor sleep quality in hospitals. Noise was identified as the primary cause of sleep disruption, with sound levels exceeding the recommendations by the WHO in all wards measured. Additionally, clinical interventions and acute conditions were identified as key disruptive factors for sleep in the hospital. Data analysis demonstrated a moderate positive association between patients’ sleep quality and their overall hospital experience.

### Interpretation within the context of the wider literature

The findings of this study align with previous research showing that 30–50% of patients experience significantly disrupted sleep in hospitals [1, 8]. The results of our study were mixed, however, with 38% of patients also reporting ‘good’ or ‘very good’ sleep quality in the hospital. Noise was identified as the primary cause of sleep disruption in the hospital, which is also consistent with previous research [1, 18]. Notably, results identified that peak sound levels exceeded 100 dB in all shared rooms, which is comparable to the sound levels produced by a lawn mower [19].

Furthermore, the results revealed a significant difference in sleep quality between patients in shared and single rooms, with sleep quality considerably worse for those in shared rooms. Differences in peak sound levels between single and shared rooms were more pronounced than average sound levels. These results align with previous research, which also concluded that average sound levels in single rooms were not significantly different from shared rooms [20]. However, solely measuring average sound levels may not be sufficient to identify the problematic nature of noise in hospitals. Therefore, it is necessary to evaluate both average and peak sound levels to comprehensively assess the hospital environment. In addition, the variance in patient-reported sleep quality measures between those in single and shared rooms might be attributed to the greater number and intensity of sound peaks in shared rooms. Consequently, these elevated sound levels could be a key factor in exacerbating sleep disturbances and diminishing sleep quality for patients in shared rooms.

### Implications for policy, practice, and research

The findings of this study highlight the significant and frequently underestimated safety consequences of sleep disturbances among patients in acute hospital environments. To address this, policymakers might consider implementing regulations that limit sound levels across the hospital, especially during night-time hours. The increased sound disturbances identified in shared rooms could also inform policies pertaining to hospital design, possibly prompting a shift towards single-room accomodation to promote better sleeping conditions for patients. In terms of practical implementation, patient-reported measurement tools (like the sleep disruption in hospitals survey) may be useful to provide information to guide quality improvement interventions. Also, the introduction of staff education programs focused on sleep health could be beneficial, which may increase awareness of the detrimental effects of noise and other interruptions on patients’ sleep and recovery. Furthermore, aligning nursing interventions with strategies that promote sleep, such as consolidating nursing activities to reduce sleep interruptions, could reduce sleep disturbances for patients. With regard to research implications, this study highlights the persistent challenge of noise in hospital environments, advocating for additional research to identify specific sources of ward noise. These findings indicate significant differences in sleep measures between patients in shared and single rooms, which was reflected in both qualitative and quantitative methods. Future research may extend the period of evaluation, including day-time sound measurement, across a range of healthcare environments, including non-acute settings.

### Strengths and limitations

This research demonstrates notable strengths through its implementation of a multimethod approach, effectively combining patient-reported measures with quantitative evaluation through noise measurement. The involvement of sleep professionals associated with the Sleep Health Foundation in guiding and reviewing the study further bolsters its credibility. Additionally, the study evaluated sleep disturbances in various patient accommodation scenarios, including both single and shared rooms, which provides significant insights to enhance the design of healthcare facilities.

However, the study does have certain limitations that warrant acknowledgment. Firstly, the exclusive provision of the survey in English may have introduced response bias, indicating the need for more diverse language options in future research. Secondly, data was collected from a single public hospital in Australia; hence, it would be beneficial to measure sleep quality and sound levels across a wider range of healthcare environments, such as private hospitals and aged care settings. This research marks the first-time use of the survey tool in a hospital setting, so further exploration and replication may be conducted to validate its effectiveness. Sound level measurement was conducted on a single night; hence, future research may investigate sound levels over consecutive days to provide a more comprehensive understanding of noise in hospital environments. The survey did not identify the specific sources of hospital noise; therefore, subsequent research may identify and address the primary causes of hospital noise. Also, multivariable analyses were not conducted; therefore, the analysis did not include control for factors that may influence sleep and other outcomes. Finally, it is noted that the associations identified may be bidirectional.

## Conclusion

This research provided significant insights into the factors contributing to sleep disturbances in hospital environments, including an examination of the differences in sleep quality as reported by patients in single and shared rooms. Sleep disturbances were evaluated using both patient-reported measures and environmental noise assessments. A significant number of patients were dissatisfied with their sleep experience in the hospital, with 40% of respondents rating their sleep quality as poor or very poor. Notably, a moderately positive correlation was identified between sleep quality and hospital experience ratings. Among the various factors assessed, noise was identified as the biggest disruptor to sleep in hospitals, with one in five patients categorizing noise as ‘very disruptive’. Additionally, acute medical conditions and nursing interventions were recognized as significant contributors to sleep disturbances. Sound-level measurement identified higher average and peak sound levels in shared rooms, along with a greater number of sound-level peaks, compared to single rooms, which could exacerbate sleep disruptions for patients. These findings from this study highlight the need for effective management of sound levels in hospitals, particularly in shared rooms, to enhance patient safety and experience.

## Supplementary Material

mzae042_Supp

## Data Availability

The data that support the findings of this study are available from the corresponding author, C.A., upon reasonable request.
